# Obstructive sleep apnoea and 12-month weight loss in adults with class 3 obesity attending a multidisciplinary weight management program

**DOI:** 10.1186/s12902-021-00887-3

**Published:** 2021-11-14

**Authors:** Sophie Kobuch, Fiona Tsang, Ritesh Chimoriya, Daniel Gossayn, Sarah O’Brien, Javeria Jamal, Leon Laks, Abd Tahrani, Nic Kormas, Milan K Piya

**Affiliations:** 1 School of Medicine, Western Sydney University, Campbelltown, New South Wales Australia; 2 South Western Sydney Metabolic Rehabilitation and Bariatric Program, Camden and Campbelltown Hospitals, Campbelltown, New South Wales Australia; 3 Australian Sleep Diagnostics, Campbelltown, New South Wales Australia; 4 Institute of Metabolism and Systems Research, University of Birmingham, Birmingham, UK; 5 Centre for Endocrinology, Diabetes and Metabolism (CEDAM), Birmingham Health Partners, Birmingham, UK; 6 Department of Diabetes and Endocrinology, University Hospitals Birmingham NHS Trust, Birmingham, UK; 7 Macarthur Clinical School, Western Sydney University, Parkside Crescent, 2560 Campbelltown, NSW Australia

**Keywords:** Obstructive sleep apnoea, Continuous positive airway pressure (CPAP), Weight management, Class 3 obesity

## Abstract

**Background:**

Although there is a strong association between obesity and obstructive sleep apnoea (OSA), the effects of OSA and CPAP therapy on weight loss are less well known. The aim of this study in adults with class 3 obesity attending a multidisciplinary weight management program was to assess the relationship between OSA and CPAP usage, and 12-month weight change.

**Methods:**

A retrospective cohort study of all patients commencing an intensive multidisciplinary publicly funded weight management program in Sydney, Australia, between March 2018 and March 2019. OSA was diagnosed using laboratory overnight sleep studies. Demographic and clinical data, and use of CPAP therapy was collected at baseline and 12 months. CPAP use was confirmed if used ≥4 h on average per night on download.

**Results:**

Of the 178 patients who joined the program, 111 (62.4 %) completed 12 months in the program. At baseline, 63.1 % (*n*=70) of patients had OSA, of whom 54.3 % (*n*=38) were using CPAP. The non-OSA group had more females compared to the OSA with CPAP group and OSA without CPAP group (90.2 % vs. 57.9 % and 62.5 %, respectively; *p*=0.003), but there were no significant baseline differences in BMI (50.4±9.3 vs. 52.1±8.7 and 50.3±9.5 kg/m^2^, respectively; *p*=0.636). There was significant weight loss across all three groups at 12 months. However, there were no statistically significant differences across groups in the percentage of body weight loss (OSA with CPAP: 6.3±5.6 %, OSA without CPAP: 6.8±6.9 %, non-OSA: 7.2±6.5 %; *p*=0.844), or the proportion of patients who achieved ≥5 % body weight loss (OSA with CPAP: 57.9 %, OSA without CPAP: 59.4 %, non-OSA: 65.9 %; *p*=0.743). In patients with T2DM, there was a significant reduction in HbA1c from baseline to 12 months (7.8±1.7 % to 7.3±1.4 %, *p*=0.03), with no difference between groups (*p*=0.997).

**Conclusions:**

This multidisciplinary weight management program resulted in significant weight loss at 12 months, regardless of OSA diagnosis or CPAP use in adults with class 3 obesity. Larger studies are needed to further investigate the effects of severity of OSA status and CPAP use in weight management programs. Until completed, this study suggests that the focus should remain on implementing lifestyle changes and weight management regardless of OSA status.

## Background

Obesity is a complex medical condition which is impacted by a variety of factors such as genetics, environment, culture and individual lifestyle [1]. Class 3 obesity refers to individuals with a body mass index (BMI) greater than 40 kg/m^2^ and is linked to increased mortality as well as risk of a range of health concerns including hypertension, type 2 diabetes (T2DM), coronary artery disease, and obstructive sleep apnoea (OSA) [1, 2]. The relationship between class 3 obesity and these conditions is often reciprocal with evidence suggesting an interplay between obesity, T2DM and OSA, which results in an increase in incidence and severity of all three [3–5].

OSA is a condition characterized by recurrent loss of tone of the upper airway resulting in hypopnoea (partial obstruction) and apnoea (complete obstruction) causing hypoxia and subsequent waking from sleep [6]. It is a significant contributor to motor vehicle accidents when not treated [7], and has been shown to exacerbate long-term diabetes complications such as cardiovascular disease (CVD), neuropathy, retinopathy and nephropathy [8–11]. While polysomnography is the “gold standard” for OSA diagnosis, it is often not widely used due to its costs and reduced availability. In virtue of this, questionnaires are valuable for the screening of the disease. These include the Epworth Sleepiness Scale (ESS), the Berlin Questionnaire (BQ) as well as the STOP-Bang Questionnaire and the STOP Questionnaire [12, 13]. First line treatment for moderate to severe OSA is continuous positive airway pressure (CPAP) [14]. Unfortunately, compliance with CPAP at one year is estimated to be as low as 50 % due to discomfort experienced by patients, challenging treatment titration processes, cost of CPAP and other psychological factors [4, 15–17].

Obesity can affect the pathogenesis of OSA in multiple ways, including upper airway fat deposition and muscle impairment, pressure from abdominal fat, leptin resistance and increased inflammatory state [18]. Significant weight loss can also reduce symptoms of OSA [14, 19, 20], but long term weight loss which is sufficient enough to reduce the severity of OSA in individuals with class 3 obesity is difficult to achieve and maintain, particularly without bariatric surgery [21–23]. On the other hand, the role of OSA on weight gain and the development of obesity is more controversial. Several factors could affect the pathogenesis of obesity in people with OSA, including daytime sleepiness and associated reduced physical activity as well as sleep deprivation affecting hunger and satiety hormones, and altering brain activity in the reward centre [18, 24, 25]. This controversy is not helped by the fact that a meta-analysis of 25 randomised trials suggested an increase in weight over time for people with OSA using CPAP therapy [26]. One explanation for this is the reduction in sympathetic activation and leptin levels following CPAP treatment which could lead to weight gain [27, 28]. However, the trials included in the meta-analysis were not from weight management programs, nor in people with class 3 obesity. It is important to understand the impact of OSA and CPAP on weight outcomes within the context of a weight management program. Therefore, the aims of this study in people with class 3 obesity were:

1. To compare weight loss over 12 months between patients with and without OSA.
2. Among patients with OSA, to compare weight loss over 12 months between those who used CPAP for ≥4 h/night and those who did not.
3. To determine whether the baseline Epworth Sleepiness Scale score is associated with weight loss at 12 months.

## Methods

This was a retrospective cohort study conducted in a publicly funded, hospital based, intensive, multidisciplinary weight management program in greater Sydney, which has been previously described [29, 30]. Patients included in the study had been enrolled in the program between March 2018 and March 2019, were over 18 years of age, had a BMI ≥40 kg/m^2^, and had at least 12 months follow up in the program. Patients attended two education sessions prior to their first medical appointment with a physician. The multidisciplinary team is comprised of endocrinologists, gastroenterologist, diabetes specialist nurse (educator), dietitians, clinical psychologists, physiotherapists, and a psychiatrist, with patients being reviewed by a local sleep physician for OSA screening and CPAP therapy use/compliance. Patients are provided individualised care every 4-12 weeks depending on clinical need and availability. The study was approved by the South West Sydney Local Health District (SWSLHD) Human Research Ethics Committee as a Quality Assurance Project (Reference: CT22_2018).

### Measurements

Electronic and paper records were reviewed to obtain demography, anthropometry and medical details including medications, blood results and sleep data. At each visit, weight was recorded on the same scale. Weight loss at 12 months was measured as both kilograms lost and as the percentage change from baseline weight. Every patient completed the Epworth Sleepiness Scale (ESS) [13] and Berlin Questionnaire at baseline, and the ESS at the 12 month visit, regardless of previous diagnosis of OSA or use of CPAP. The ESS is a self-administered questionnaire which measures a person’s general level of daytime sleepiness during commonly encountered situations. OSA was confirmed with diagnostic laboratory sleep studies before or soon after program commencement. CPAP compliance was considered if treated with an average of ≥4 h/night on download. Any data collected between 11 and 13 months was considered for the 12-month time frame due to variations in follow up appointments. Data was de-identified before analysis.

### Data analysis

Differences in baseline between three groups on the basis of OSA diagnosis and CPAP use: OSA with CPAP, OSA without CPAP, and non-OSA groups, were evaluated for patients completing 12-month follow up. Kolmogorov–Smirnov test and Shapiro–Wilk test were performed to test normality of the data. Analysis of variance (ANOVA) was conducted for continuous variables with normal distribution, Kruskal–Wallis test for continuous data without normal distribution, and Pearson’s chi-square test for categorical variables. Analysis of covariance (ANCOVA) was conducted to evaluate the changes across the three groups and adjust for the covariates. Paired sample t-tests and Wilcoxon signed rank test were used to test for differences between baseline and 12 months within each group. Weight loss at 12 months was reported as percentage body weight loss. Relationships between continuous variables were determined through the Pearson’s correlation coefficient. *P*<0.05 was considered statistically significant. All statistical analyses were performed with the Statistical Package for Social Sciences (SPSS) Version 25.

## Results

**Fig. 1:**
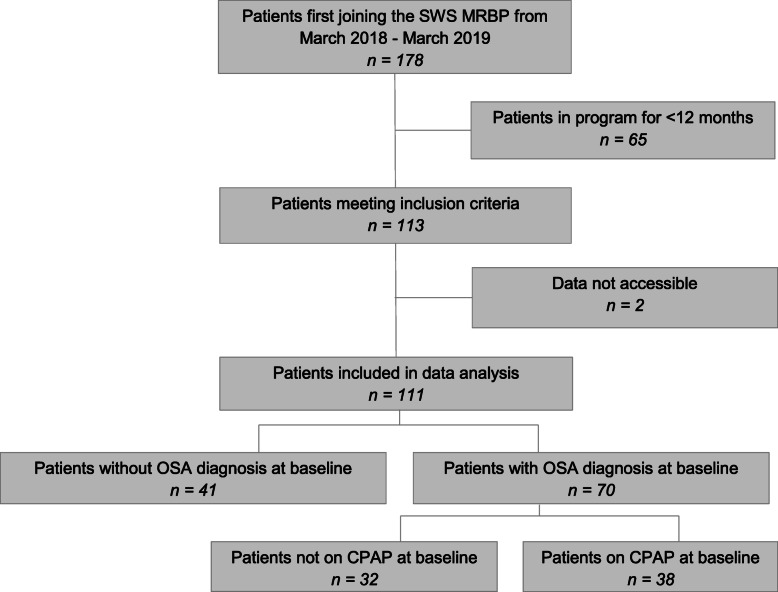
Participant. SWS MRBP = South Western Sydney Metabolic Rehabilitation and Bariatric Program

A total of 178 patients joined the South Western Sydney Metabolic Rehabilitation and Bariatric Program between March 2018 and March 2019. Of those, 111 (62.4 %) attended follow-up at 12 months and had data available as shown in Fig. 1.

### Baseline characteristics

The baseline data of patients were categorised into three groups on the basis of OSA diagnosis and CPAP use: OSA with CPAP, OSA without CPAP, and non-OSA groups (Table 1). OSA had been diagnosed at baseline in 63.1 % (*n*=70) of patients, of whom 54.3 % (*n*=38) were using CPAP. The non-OSA group had more females compared to OSA with CPAP and OSA without CPAP groups (90.2 % vs. 57.9 % and 62.5 %, respectively; *p*=0.003). There were no significant baseline differences in age (50.6 ± 14.7 vs. 57.0 ± 12.4 and 53.5 ± 10.9 years, respectively; *p*=0.100), weight (134.9 ± 29.2 vs. 148.1 ± 31.3 and 140.4 ± 26.6 kg, respectively; *p*=0.137), or BMI (50.4 ± 9.3 vs. 52.1 ± 8.7 and 50.3 ± 9.5 kg/m^2^, respectively; *p*=0.636) across all groups. The OSA without CPAP group had a higher proportion of participants with gastro-oesophageal reflux disease (GORD) compared to OSA with CPAP and non-OSA groups (62.5 % vs. 34.2 % and 43.9 %, *p*=0.039). However, there were no significant differences between the three groups in the proportion of patients who had hypertension, type 2 diabetes, dyslipidaemia, cardiovascular disease, or non-alcoholic fatty liver disease, with no differences in the baseline lipid profile and baseline ALT as depicted in Table 1.

**Table 1:** Baseline characteristics of all participants, and across the three groups: OSA with CPAP, OSA without CPAP, and non-OSA

*Variable* Mean±SD or *n*(%)	*OSA (n=70)*		*Non-OSA (n=41)*	p value^*^
	***OSA with CPAP (n=38)***	***OSA without CPAP (n=32)***		
Age (years)	57.0 ± 12.4	53.5 ± 10.9	50.6 ± 14.7	0.100
Female	22 (57.9 %)	20 (62.5 %)	37 (90.2 %)	**0.003**
Caucasian	24 (63.2 %)	23 (71.9 %)	33 (80.5 %)	0.230
Not in paid employment	27 (71.1 %)	17 (53.1 %)	20 (48.8 %)	0.112
Weight (kg)	148.1 ± 31.3	140.4 ± 26.6	134.9 ± 29.2	0.137
BMI (kg/m^2^)	52.1 ± 8.7	50.3 ± 9.5	50.4 ± 9.3	0.636
Hypertension	28 (73.7 %)	23 (71.9 %)	24 (58.5 %)	0.294
T2DM	26 (68.4 %)	20 (62.5 %)	23 (56.1 %)	0.528
Dyslipidaemia	28 (73.7 %)	25 (78.1 %)	25 (61.0 %)	0.240
Cardiovascular disease	9 (23.7 %)	6 (18.8 %)	5 (12.2 %)	0.441
Non-Alcoholic Fatty liver disease (NAFLD)	5 (13.2 %)	7 (21.9 %)	6 (14.6 %)	0.579
Gastro-oesophageal Reflux Disease (GORD)	13 (34.2 %)	20 (62.5 %)	18 (43.9 %)	**0.039**
Total Cholesterol (mmol/L)	4.5 ± 1.1	4.4 ± 0.9	4.4 ± 1.2	0.880
Triglycerides (mmol/L)	1.9 ± 1.5	1.8 ± 0.8	1.8 ± 0.8	0.845
LDL Cholesterol (mmol/L)	2.5 ± 1.0	2.5 ± 0.7	2.4 ± 1.2	0.925
HDL Cholesterol (mmol/L)	1.1 ± 0.3	1.1 ± 0.3	1.2 ± 0.3	0.642
ALT (IU/L)	33.4 ± 23.5	27.8 ± 13.4	30.2 ± 14.5	0.447
Epworth Sleepiness Scale score	7.7 ± 5.3	8.5 ± 4.8	5.8 ± 5.1	0.078
Berlin Score	2.5 ± 0.7	2.4 ± 0.7	2.3 ± 0.7	0.532

^*^p-values reflects the difference across three groups.

At baseline, 34 patients (89.5 %) in the OSA with CPAP group, 28 patients (87.5 %) in the OSA without CPAP group, and 32 patients (78.0 %) in the non-OSA group completed the Berlin questionnaire. The majority of patients had a high-risk Berlin score in all three groups, with no significant differences between the three groups (*p*=0.532). All participants completed the ESS questionnaire at baseline, with no statistically significant differences in the baseline ESS score when compared across all three groups.

### Baseline vs. 12 months

#### Changes in Weight and ESS score

**Fig. 2:**
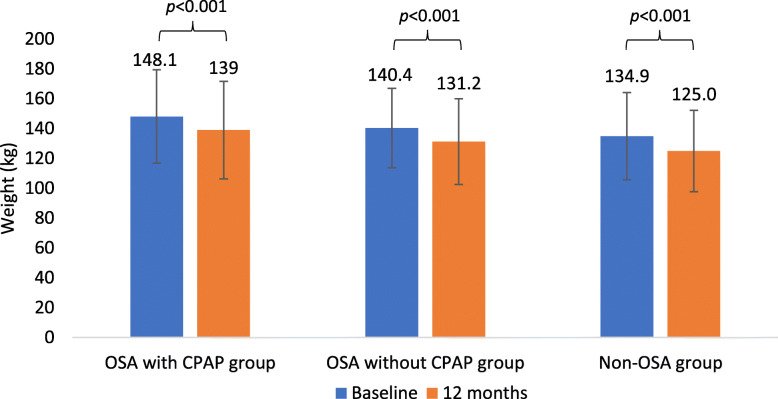
Weight change from baseline to 12 months across the three groups. (Data expressed as mean ± SD in the graphs)

Significant weight loss was observed across all three groups at 12 months (Fig. 2). However, there were no statistically significant differences in weight change at 12 months between the three groups (Table 2). Having a diagnosis of OSA did not affect percentage body weight loss at 12 months (*p*=0.610), nor the proportion of patients who achieved 5 % weight loss (*p*=0.449), nor who achieved 10 % weight loss (*p*=0.897). There was also no statistically significant association of CPAP use with weight loss at 12 months (*p*=0.774).

There was a significant reduction in the ESS score from baseline to 12 months in all three groups– OSA with CPAP: 7.7 ± 5.3 vs. 4.2 ± 5.6, *p*<0.001; OSA without CPAP: 8.5 ± 4.8 vs. 6.0 ± 6.2, *p*<0.034; non-OSA: 5.8 ± 5.1 vs. 2.7 ± 3.1, *p*<0.001. However, there were no significant differences in change in ESS score at 12 months across the three groups– OSA with CPAP: 3.5 ± 5.9, OSA without CPAP: 2.5 ± 6.3, non-OSA: 3.1 ± 5.1; *p*=0.753 (Table 2). There was also no significant correlation between percentage weight loss and change in ESS score at 12 months (*r*=0.08, *p*=0.398).

**Table 2:** Changes in weight, lipid profile and Epworth Sleepiness Scale score between baseline and 12 months across the three groups of participants: OSA with CPAP, OSA without CPAP, and non-OSA

*Variable* Mean±SD or *n*(%)	*OSA (n=71)*		*Non-OSA (n=41)*	Unadjusted p value^**^	*P-value adjusted for age and baseline weight*
	***With CPAP (n=38)***	***Without CPAP (n=32)***			
**Weight Change at 12 months**					
***Weight loss (in kg)***	9.1 ± 7.9	9.2 ± 9.9	9.9 ± 9.6	0.903	0.172
***Percentage of body weight loss***	6.3 ± 5.6	6.8 ± 6.9	7.2 ± 6.5	0.844	0.149
***More than 5 % body weight loss***	22 (57.9 %)	19 (59.4 %)	27(65.9 %)	0.743	0.361
**Change in Lipid profile**					
***Cholesterol (mmol/L)***	0.8 ± 1.1	0.1 ± 1.3	-0.3 ± 1.6	**0.010**	**0.011**
***Triglyceride (mmol/L)***	0.3 ± 1.8	-0.2 ± 1.4	-0.5 ± 2.6	0.318	0.596
***HDL (mmol/L)***	0.1 ± 0.4	0.1 ± 0.5	0.0 ± 0.4	0.912	0.877
***LDL (mmol/L)***	0.8 ± 1.1	0.4 ± 1.4	0.0 ± 1.0	0.140	0.280
**Change in Epworth Sleepiness Scale (ESS) score at 12 months**					
***ESS Score***	3.5 ± 5.9	2.5 ± 6.3	3.1 ± 5.1	0.753	0.399

#### Changes in lipid profile

**Fig. 3:**
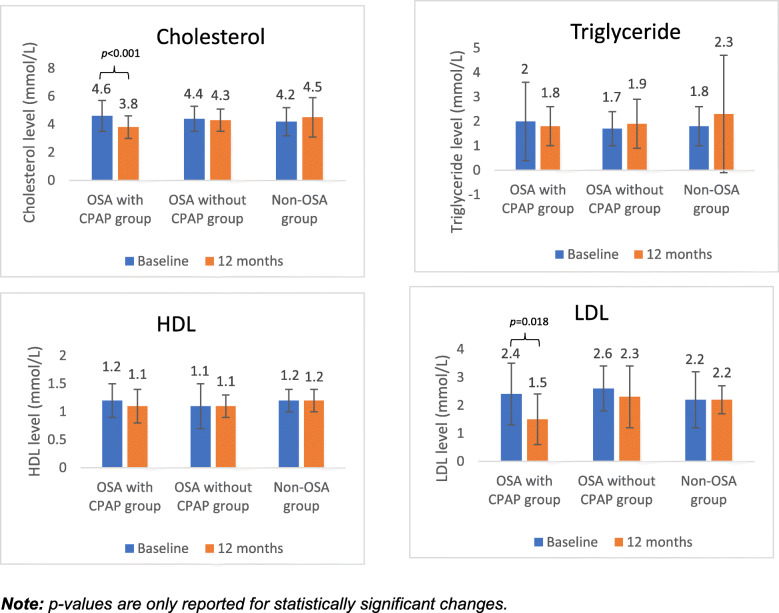
Changes in lipid profiles from baseline to 12 months across three groups

There was a significant difference in the decrease in cholesterol at 12 months across the three groups (*p*=0.010), which remained significant when adjusted for age and baseline weight (Table 2). Furthermore, it was revealed that the reduction in cholesterol at 12 months was statistically significant in the OSA with CPAP group (baseline: 4.6 ± 1.1 mmol/L vs. 12 months: 3.8 ± 0.8 mmol/L, *p*<0.001), but not in the OSA without CPAP group (baseline: 4.4 ± 0.9 mmol/L vs. 12 months: 4.3 ± 0.9 mmol/L, *p*=0.342), nor in the non-OSA group (baseline: 4.2 ± 1.0 mmol/L vs. 12 months: 4.5 ± 1.4 mmol/L, *p*=0.368) as presented in Fig. 3. There was no significant difference across the three groups in the change in triglyceride, HDL and LDL at 12 months as demonstrated in Table 2. However, there was a significant decrease in LDL at 12 months in the OSA with CPAP group (2.4 ± 1.1 mmol/L vs. 1.5 ± 0.9 mmol/L, *p*=0.018), but not in the OSA without CPAP group (2.6 ± 0.8mmol/L vs. 2.3 ± 1.0mmol/L, *p*=0.733), nor in the non-OSA group (2.2 ± 1.0mmol/L vs. 2.2 ± 0.5mmol/L, *p*=0.924) as presented in Fig. 3. There were no significant changes in HDL and triglyceride in all three groups between baseline and 12 months. There was no significant difference in the overall number of patients on cholesterol medication at baseline compared to 12-months (58.5 % vs. 64.0 %, *p*=0.408), and no significant difference within each group: OSA with CPAP group (65.8 % vs. 71.1 %, *p*=0.621), the OSA without CPAP group (59.4 % vs. 65.6 %, *p*=0.605), or in the non-OSA group (51.2 % vs. 56.1 %, *p*=0.658).

#### Patients with diabetes

There was no significant difference in the percentage of patients in each group who had a diagnosis of T2DM at baseline (OSA with CPAP: 68.4 %, OSA without CPAP: 62.5 %, non-OSA: 56.1 %; p=0.528). Of the patients with T2DM, 38.4 % in the OSA with CPAP group, 30 % in the OSA without CPAP group and 30.4 % in the non-OSA group were on insulin at baseline. There was no significant difference in the proportion across the three groups (*p*=0.778), with an average daily insulin dose of 173.9 ± 106.9 units for OSA with CPAP, 109.7 ± 56.7 units for OSA without CPAP, and 102.1 ± 54.0 units for the non-OSA group (*p*=0.366).

At 12 months, among patients with T2DM, 42.3 % in the OSA with CPAP group, 30 % in the OSA without CPAP group and 26.1 % in the non-OSA group were on insulin (*p*=0.448). The average daily insulin dose was 106.9 ± 65.6 U for OSA with CPAP, 56.7 ± 56.3 U for OSA without CPAP, and 54.0 ± 25.9 U for non-OSA group (*p*=0.370). From baseline to 12 months, there was no significant difference in the proportion of patients on insulin across all three groups. There were statistically significant reductions in insulin dose across two groups (OSA with CPAP: *p*=0.014; non-OSA: *p*=0.002), and non-significant reduction in insulin dose in the OSA without CPAP group (*p*=0.194). In the overall study population, there was a significant reduction in HbA1c from baseline to 12 months (7.8±1.7 % to 7.3±1.4 %, *p*=0.03), with no difference in the reduction in HbA1c across three groups (OSA with CPAP: 0.5 ± 1.5 %, OSA without CPAP: 0.5 ± 1.1 %, non-OSA: 0.6 ± 1.6 %, *p*=0.997).

## Discussion

In this single centre cohort study, individuals with class 3 obesity attending a publicly funded weight management program had significant weight loss in both those with and without OSA. There was no significant difference in weight loss between the groups, and the use of CPAP in the OSA group did not affect the 12-month weight outcomes.

Although significant, the percentage weight loss over the 12-month period in this high-risk population was modest (6-7 %), which is akin to previous studies [29–31]. The impact of OSA on obesity is not completely understood. While multiple mechanisms would logically link the sleep deprivation experienced by patients with OSA to weight gain such as reduction in physical activity and eating behaviours favouring high calorie containing foods [32, 33], as well as an activation of the hypothalamic-pituitary-adrenal axis and increased cortisol, the long term evidence of the impact of OSA on weight is sparse [34]. While the increased tiredness and sleepiness often associated with OSA would not favour successful weight loss interventions, patients who use CPAP generally report dramatic improvements in alertness, mood and quality of life, each of which may be essential in attempting to lose weight [35]. However, a systematic review of over 3000 patients from 25 randomised controlled trials suggested an increase in weight over time for people with OSA using CPAP therapy [26], that was greater with increasing baseline weight. Thus, it is perhaps not surprising that in this study, those with OSA treated with CPAP did not appear to have improved weight loss compared to those with untreated OSA. Therefore, weight loss in OSA patients seems to be multifactorial, and cannot at this stage be solely achieved with CPAP therapy. Indeed, multiple factors have been hypothesised for this finding, including the reduction in sympathetic activity leading to reduced lipolysis and energy expenditure, leading to weight gain [26]. Of note, the systematic review did not evaluate the effect of CPAP on weight loss among patients following a weight management program. Furthermore, these studies did not evaluate the weight loss in people with class 3 obesity.

While there may be the preconceived notion that being diagnosed with OSA impacts on the ability for patients to lose weight, our study suggests that weight loss outcomes are similar, regardless of the presence of OSA diagnosis at baseline or the use of CPAP. This has implications for both patients and clinicians alike, in alleviating the concerns of how OSA diagnosis or use of CPAP may impact weight loss. Instead, emphasis can be placed on implementing lifestyle changes that may induce a modest amount of weight loss that can be beneficial to the metabolic risk profile of individuals with class 3 obesity [3, 36].

There are, however, undeniable benefits of CPAP therapy in improving OSA severity and thereby sleep quality on overall health. Several studies have shown a high prevalence of T2DM in patients with OSA and vice versa[37, 38], OSA being an independent risk factor for the development of T2DM [39]. We have recently demonstrated that patients with class 3 obesity had a reduction in their HbA1c by 0.47 % over 6 months in this weight management program [30]. In this study, we report no difference at baseline in the proportion of patients who have T2DM, nor in the daily dose of insulin in all three groups. However, the reduction in HbA1c was consistent with our previous study and was similar regardless of CPAP use. This supports the notion that OSA diagnosis or CPAP use may not affect glycaemic control, in the parameters of a medical weight management program in people with class 3 obesity. There are inconsistent findings in the effects of CPAP use for 1-6 months on improvement of glycaemic control [40–43]. Well-controlled experiments with compliant CPAP use for 8 h a night have shown to improve glucose metabolism in one to two weeks [44, 45]. However, the largest study to date which included close to 900 participants followed over 4.3 years showed no significant difference between the CPAP and usual care groups in their serum glucose or HbA1c [46]. Larger studies of longer duration are needed to assess the impact of CPAP on the prevention of T2DM.

People with OSA on CPAP had a significant drop in the total and LDL cholesterol levels at 12 months compared to baseline, whereas there was no significant change in those with OSA not on CPAP, nor in those without OSA. This is interesting, given that there were no differences in use of cholesterol lowering medications overall or in any of the groups from baseline to 12 months. While clear evidence of the benefits of CPAP use on reducing cholesterol levels is lacking, it has been postulated that CPAP may improve patients’ lipid profile by reducing the intermittent hypoxia, as well as through increased physical activity [47]. These findings should be confirmed in larger trials, and possible mechanisms warrant further investigation.

Hypertension is another well-known risk factor for cardiovascular disease and overall mortality. Blood pressure decreases during sleep, which is likely related to decreases in sympathetic output. A lack of this “nocturnal dipping”, which is inevitably the case in patients with OSA, has been associated with a greater risk of cardiovascular mortality [48]. Even a 10mm Hg increase in mean night-time systolic blood pressure has been shown to be associated with a 21 % increase in cardiovascular mortality [49]. Multiple large studies have shown that people who sleep less than five hours per night have a greater risk of hypertension than those sleeping more than 7 h per night [50, 51]. Therefore, it seems logical that improving sleep would reduce the incidence of hypertension in patients with OSA. A retrospective study in patients with T2DM and newly diagnosed OSA, showed that the use of CPAP for 9-12 months lead to a significant reduction in blood pressure [52]. Thus, improving OSA severity and thereby increasing sleep quality has the potential to substantially improve cardiovascular morbidity and mortality. Hence, it is encouraging to see a significant reduction in the Epworth Sleepiness Scale score over a 12-month period in all three groups in our study. In addition to the benefits of reduced sleepiness on driving and day to day functioning, it may be that the benefits of the reduction in sleepiness on weight outcomes are seen later than at the 12-month period.

As many patients in this study, and people with class 3 obesity in general, would often meet the criteria for bariatric surgery, it is of utmost importance to optimise pre-operative health, including OSA severity, to minimise peri-operative complications. Indeed, there is a positive association between OSA severity and post-operative risk, including increased risk of shock and cardiac arrest [53]. Therefore, improving OSA status prior to bariatric surgery may prevent peri-operative complications. In virtue of this, clinicians and allied health professionals should focus on emphasising improvements in OSA status, and herewith CPAP compliance, in addition to weight loss per se, in patients with class 3 obesity.

The main strength of this study is that it is based in a real-world weight management program, that included all patients from a publicly funded metabolic program within a hospital service. These findings can therefore inform other multidisciplinary medical weight management programs. Limitations are that this is a single centre with a relatively small cohort of patients, and only 12 months follow up. Multicentre studies which include large sample sizes and longer follow up would help corroborate the findings from this study. Furthermore, a larger sample size would have allowed us to assess the severity of OSA, and its impact on weight loss. Finally, reasons for why patients did or did not commence CPAP were not explored in this study. Such analysis may help identify barriers not only to CPAP use, but also to weight loss.

## Conclusions

This single centre retrospective cohort study demonstrated that the presence of OSA at baseline or the use of CPAP does not affect weight or glycaemic outcomes at 12 months in people with class 3 obesity in the context of a medical weight management program. Findings from this study suggest that the focus should remain on implementing lifestyle changes and medical weight management in people with class 3 obesity, regardless of OSA status.

## Data Availability

The datasets used and analysed during the current study are available from the corresponding author on reasonable request.

## Abbreviations

ALT: alanine aminotransferase
ANOVA: analysis of variance
ANCOVA: analysis of covariance
BMI: Body Mass Index
BQ: Berlin Questionnaire
CPAP: continuous positive air pressure
CVD: cardiovascular disease
ESS: Epworth Sleepiness Scale
HbA1c: glycosylated hemoglobin
HDL: high density lipoprotein
IU/L: international units per litre
kg: kilogram
kg/m^2^: kilogram per meter squared
LDL: low density lipoprotein
mmol/L: millimoles per litre
OSA: obstructive sleep apnoea
SD: standard deviation
SES: socioeconomic status
SPSS: Statistical package for social sciences
SWSLHD: South West Sydney Local Health District
SWS MRBP: South Western Sydney Metabolic Rehabilitation and Bariatric program
T2DM: type 2 diabetes mellitus
U: units
